# Evaluating anaesthetic impacts on rainbow trout mucus biomarkers: towards sustainable aquatic animal welfare

**DOI:** 10.1007/s10695-025-01602-y

**Published:** 2025-11-15

**Authors:** M. Tejero, L. Fernandez-Alacid, I. Sanahuja, A. Vallejo-Castaño, C. Balsalobre, C. Madrid, A. Ibarz

**Affiliations:** 1 Department of Cell Biology, Physiology and Immunology, Faculty of Biology, University of Barcelona, Avda. Diagonal 643, 08028 Barcelona, Spain; 2 Department of Genetics, Microbiology and Statistics, Faculty of Biology, University of Barcelona, Avda. Diagonal 643, 08028 Barcelona, Spain

**Keywords:** Anaesthesia, MS-222, Clove oil, Skin mucus, Rainbow trout

## Abstract

Anaesthesia is a necessary step during fish manipulation. Tricaine methane-sulfonate (MS-222) is the most commonly used anaesthetic in experimental trials; however, its use in food fish production is strictly regulated. This study aimed to evaluate clove oil, less persistent and authorized in European countries, as an alternative to MS-222 for skin mucus analyses in aquaculture settings. For this purpose, rainbow trout individuals from a commercial factory were sedated with clove oil or MS-222. The concentration of protein, glucose, lactate, and cortisol and the antibacterial activity were measured in both skin mucus and plasma. Additionally, FRAP, total protease activity, and lysozyme activity were also analysed for mucus samples. Recovery times differed significantly between treatments, being approximately four times longer for clove oil (at the farm’s standard dose) than for MS-222 (at the standard laboratory dose). However, none of the stress-related biomarkers in mucus were affected by the anaesthetic treatment, whereas plasma from clove oil-treated fish showed increased lactate and reduced cortisol levels. The in vitro bacterial growth inhibition assay using mucus and plasma provides a reliable and rapid method for assessing fish innate immunity. No significant differences were observed between treatments against any of the bacterial strains tested. Overall, the findings strongly endorse the use of skin mucus as an effective method for studying and monitoring trout in aquaculture settings.

## Introduction

During their life cycle, farmed and experimental fish are subjected to different procedures such as netting, transport, weighing, vaccination, and research trials, which may stress the animals or even compromise their health by the physiological changes and possible injuries produced while handling (Carter et al. 2011; Zahl et al. 2012). Therefore, anaesthesia is required during fish manipulation in both aquaculture and research to maintain animal welfare. Indeed, the European Parliament and Council of the European Union (2010), on the protection of animals used for scientific purposes, states that anaesthesia shall be mandatory for any regulated procedure unless the anaesthetic procedure itself interferes with the scientific objectives or is judged to be more traumatic to the animal than the procedure itself (Schroeder et al. 2021). Moreover, the choice of anaesthetic agent can influence physiological responses, which is particularly relevant for researchers conducting experimental trials in fish physiology.

Although a wide range of anaesthetic agents for fish have been described in the last century, tricaine methane-sulfonate (TMS or MS-222) has become one of the most used since its introduction in 1967 (Topic Popovic et al. 2012). MS-222 is the only anaesthetic licensed for food fish production in some countries such as the USA and Canada (Zahl et al. 2012), and its properties, mechanism of action, metabolism, and physiological effects have been exhaustively reviewed. However, MS-222 is known to be hazardous for humans in case of inhalation and skin contact and to potentially contaminate surrounding water ecosystems (Collymore et al. 2016; Ferreira et al. 2017). Consequently, personnel handling MS-222 must follow strict safety protocols that consider both human health and environmental risks, factors that are particularly challenging to manage in large-scale aquaculture operations or field studies involving wild fish populations. Additionally, any contaminated water resulting from its use must be properly treated to prevent environmental harm. Moreover, the European regulation establishes a withdrawal period of 21 days between MS-222 use in food fish and its commercialization (Jerez Cepa et al. 2019), hampering its use for fish management in farms. The strict European legislation on MS-222 usage has created a rift between aquaculture and laboratory research practices, where this agent is widely established. To bridge this gap, researchers should consider alternative anaesthetic agents, such as clove oil, which has a regulated withdrawal period of 24 h in European countries and has a significantly lower environmental impact.

Clove oil is a natural essential oil derived from clove trees (*Eugenia aromatic)*, a mixture of several compounds, with eugenol being the primary active ingredient (Anderson et al. 1997). It represents a nontoxic, rapidly metabolized, cheaper, less persistent, and easier-to-handle anaesthetic agent when compared to other immersion drugs (Keene et al. 1998). Therefore, several studies have investigated the potential of clove oil as a viable alternative to MS-222 and other chemical anaesthetics in fish physiology and aquaculture research. These investigations have primarily focused on comparing the anaesthetic effects of clove oil to established agents (fish behaviour, swimming performance, lethal toxicity, etc.) and concluded that clove oil results in similar responses and even more rapid induction times and consistent anaesthesia when compared to MS-222 (Anderson et al. 1997; Keene et al. 1998; Munday & Wilson 1997; Neiffer & Stamper 2009; Park 2019). The difference in induction and recovery times is particularly relevant, as it may condition the choice of anaesthetic depending on the specific experimental procedure being conducted. For instance, studies requiring minimal physiological disturbance may benefit from faster-acting agents with quick recovery, whereas longer procedures might prioritize stability and duration of anaesthesia. Literature also addressed the impacts on different physiological blood parameters and found no significant differences between fish anaesthetized with clove oil or MS-222 for most of them (Cho & Heath 2000; Holloway et al. 2004; Sladky et al. 2001; Wagner et al. 2003). Based on these results, it can be concluded that clove oil presents a compelling alternative to MS-222 for research involving fish internal physiology and related trials.

Nowadays, there is a growing interest within the scientific community regarding fish welfare for research and commercial purposes. Current legislation on animal experimentation is largely guided by the principles of the 3Rs (replacement, reduction, and refinement) to minimize pain and suffering in research animals (Sloman et al. 2019). To improve refinement, many recent studies have focused on the development of non-invasive methods for monitoring the physiological status and welfare of fish (Barreto et al. 2022). One of the most promising non-invasive or minimally invasive approaches involves the study of fish skin mucus. This mucus, secreted by epidermal cells, serves multiple crucial functions and acts as the first line of defence against a wide variety of environmental conditions (Esteban 2012; Sanahuja & Ibarz 2015; Shephard 1994; Subramanian et al. 2007). Fish skin mucus serves as a semipermeable and dynamic barrier, adapting its properties and composition in response to various environmental challenges. The mucus composition can change significantly in response to different challenges, such as dietary conditions (Fernández-Alacid et al. 2018, 2019b; Firmino et al. 2021; Herrera et al. 2020; Hoseinifar et al. 2017a, 2017b) and chronic and acute stressors (Fernández-Alacid et al. 2019b, 2019a; Herrera et al. 2020; Ordóñez-Grande et al. 2020; Sanahuja et al. 2019b). This adaptability makes mucus an invaluable indicator of fish health and stress levels, and consequently, skin mucus analysis has emerged as a powerful and non-invasive tool that allows fish recovery after sampling. Recovering the fish after sampling is not only valuable for the fish production industry, as it avoids production losses, but also for field studies involving endangered species, where non-lethal methods are essential for conservation-focused research.

In research, the process of anaesthetizing fish typically involves immersion in a solution containing the anaesthetic agent. The anaesthetic enters the bloodstream and central nervous system through the gills, accessory respiratory organs, and/or the skin (Neiffer & Stamper 2009), thus implying direct contact between the anaesthetic agent and fish skin mucus-producing cells. In this context, the different anaesthetics could potentially influence the composition and properties of the fish’s skin mucus when contacting and being absorbed by epidermal cells. Despite the widespread use of anaesthetics in aquaculture and fish research, there is limited literature available on their effects on fish skin mucus. Moreover, existing studies have been conducted under controlled laboratory conditions, highlighting the need for in situ evaluations.

In this regard, the present study aimed to evaluate the potential use of clove oil as a suitable alternative to MS-222 for research studies involving in situ farmed rainbow trout (*Oncorhynchus mykiss*). For this purpose, potential differences in anaesthetic effects, plasma and skin mucus biomarkers, and antibacterial activity were evaluated for individuals anaesthetized with MS-222 or with clove oil.

## Materials and methods

### Animals and sampling procedures

The study was conducted at a local fish farm (Piscifactoría Peramola, 25,790, Lleida), where a total of 48 rainbow trout (343.55 ± 13.46 g body weight) were randomly sampled from 3 different cages. In consecutive groups of four fish, individuals were anaesthetized in supplementary aerated 40 L tanks with either MS-222 (100 mg/L) or clove oil (0.4 mL/L) (Sigma-Aldrich, Madrid, Spain), with 24 individuals assigned to each condition. Each group was alternately exposed to one of the two anaesthetic agents to minimize handling-related differences between treatment groups. Specifically, the aim was to avoid a scenario in which fish anaesthetized with the slower-acting agent remained in the tank longer than necessary due to ongoing sampling of fish treated with the faster-acting agent. By maintaining consistent handling and sampling times, both groups could be considered sequential and comparable. Nevertheless, the potential influence of the capture process was acknowledged; accordingly, the time required to capture and transfer fish from cages to the anaesthetic tanks was measured and compared.

The MS-222 dose fell within the recommended range for rainbow trout (60–150 mg·L⁻^1^; Topic Popovic et al. 2012) and was selected based on previous studies involving similarly sized individuals (Anderson et al. 1997; Holloway et al. 2004; Pounder et al. 2018). The clove oil concentration was decided following standard farm protocols used in similar procedures for fish of comparable size.

As stated, capture time, including transport from cages to the supplementary tanks, was recorded to ensure that no significant differences arose from sampling procedures. While in the supplementary anaesthetic tanks, fish behaviour and swimming activity were observed. Time to reach full anaesthesia was carefully recorded for each fish to assess the anaesthetic efficacy of both agents. The duration of skin mucus sampling and recovery time were also measured (see Table 1).

**Table 1:** Time-lapse of the skin mucus sampling steps for the two anaesthetic treatments

	Catching time (s)	Anaesthesia time (s)	Sampling time (s)	Recovery time (s)
MS-222	32.50 ± 0.61	172.75 ± 10.70	36.13 ± 0.43	49.75 ± 4.99
Clove Oil	32.30 ± 0.98	94.00 ± 5.41*	37.20 ± 0.37	205.5 ± 25.46*

Values are expressed as mean ± SEM (standard error of mean), *n* = 12. Significant differences between treatments are indicated by * (Student’s *t*-test, *p* < 0.05)

### Sample collection and processing

Once the fish were fully anaesthetized, skin mucus was immediately collected from all individuals using a previously established method described by Fernández-Alacid et al. (2018). Briefly, sterile glass slides were gently slid along the over-lateral line of both sides of the fish in a front to caudal direction and stored in 2-mL sterile tubes. Ventral zones were excluded to avoid faecal and urogenital contamination, as well as repeatedly rubbing the skin, as it may cause epidermal injuries leading to blood and cell contamination. Mucus samples were homogenized using a sterile Teflon implement to desegregate mucus mesh before centrifugation at 14,000 × g for 15 min at 4 °C. The resultant mucus supernatants were collected, avoiding the surface lipid layer, aliquoted and stored at − 80 °C for further analysis.

Blood samples were obtained from 12 fishes per experimental condition from the caudal vein using syringes impregnated with EDTA-Li as anticoagulant. Plasma was collected after centrifugation at 12,000 × g for 5 min at 4 °C and stored at − 80 °C. These animals were then euthanized to avoid the return of injured fishes to the productive cages, according to the farm’s normative procedures. The remaining 12 fishes per condition, which were not subjected to blood sampling, were directly recovered in two 40-L additional tanks filled with oxygenated water. These tanks were used to assess the recovery time and recovery capacity for both anaesthetics.

All fish were first sampled for skin mucus prior to blood collection. As a result, all mucus samples could be considered comparable, regardless of whether the fish were subsequently subjected to blood sampling. Blood sampling was performed only on a subset of fish, and while this may have introduced additional stress, both anaesthetic groups were treated identically, ensuring valid comparisons.

The study was conducted in accordance with the local legislation and institutional requirements, following the Guiding Principles for Biomedical Research Involving Animals (EU2010/63), the guidelines of the Spanish laws (law 32/2007 and RD 53/2013). The fish manipulation and study were conducted under the authorization of the UB-Ethical Committee for Animal Experimentation and the Generalitat of Catalonia government for the use of laboratory animals (CEEA 133/23).

### Skin mucus and plasma biomarkers analysis

Glucose and lactate concentrations were determined by enzymatic colorimetric tests (LO-POD glucose and LO-POD lactate, SPINREACT®, Spain, respectively) according to the manufacturer’s instructions for plasma determinations and with slight modifications for homogenized mucus samples, as described by Fernández-Alacid et al. (2018). The optical density (OD) was determined at λ = 505 nm with a microplate reader (Infinity Pro200 spectrophotometer, Tecan, Spain) after an incubation time of 10 min at 37 °C for glucose determination and at room temperature for lactate. Values were expressed as µg metabolite/mL of skin mucus and mg metabolite/dL of plasma.

Protein concentration was determined using the Bradford assay (Bradford 1976) with bovine serum albumin (BSA; Sigma) as the standard. The OD was determined at *λ* = 596 nm with a microplate reader (Infinity Pro200 spectrophotometer, Tecan, Spain). Protein values were expressed as mg of protein/mL of skin mucus or plasma.

Cortisol levels were determined using an ELISA kit (IBL International, Germany) according to Fernández-Alacid et al. (2018). The OD was determined at λ = 450 nm with a microplate reader (Infinity Pro200 spectrophotometer, Tecan, Spain). The cortisol values were expressed as ng cortisol/mL of skin mucus or plasma.

Mucus glucose/protein, lactate/protein and cortisol/protein ratios were calculated to standardize putative dilution or concentration during mucus sampling. Glucose/lactate ratio represents an indicator of aerobic/anaerobic metabolism.

### Defensive skin mucus biomarkers

Ferric reducing antioxidant power (FRAP), lysozyme activity, and total alkaline protease activity (TPA) were analysed for mucus extracts as defensive biomarkers of fish. Anaesthesia induces several physiological and metabolic alterations, such as changes in respiratory and heart rates, that may lead to hypoxic conditions. These can disrupt cellular oxidative balance and trigger oxidative stress, activating antioxidant defence mechanisms. The activity of these mechanisms can be assessed using the FRAP method, which has previously been applied to fish skin mucus (De Mercado et al. 2018). Lysozyme and proteases are important components of the innate immune system and play a crucial role in the protective function of fish skin mucus. These biomarkers, which are simple and cost-effective to measure, have been shown to vary in response to different anaesthetic treatments (Guardiola et al. 2016; Soltanian et al. 2018).

The ferric reducing antioxidant power (FRAP) levels were determined by a colorimetric test (Ferric Antioxidant Status Detection Kit, Invitrogen). This test allows us to infer the antioxidant activity of a sample through the quantification of ferrous ions (Fe^2+^) formed from the reduction of ferric ion (Fe^3+^) after an incubation of 30 min. The assay was carried out following the manufacturer’s instructions for plasma determinations but with slight modifications (Sanahuja et al. 2019a). The OD was determined at *λ* = 560 nm with a microplate reader (Infinity Pro200 spectrophotometer, Tecan, Spain). Antioxidant values were expressed as nmol Fe^2+^/mL of skin mucus extract and then the FRAP/protein ratio was calculated.

Lysozyme activity in skin–mucus was assessed following the turbidimetric method described by Ellis (1990) with slight modifications. A standard curve was prepared by diluting chicken egg white lysozyme (Sigma) in phosphate buffer (Na_2_HPO_4_ 0.05 M, pH = 6.2). In 96-well plates, 20 µL of skin mucus samples and standard solutions were mixed with 130 µL of a *Micrococcus lysodeiktikus* (Sigma) suspension at 0.6 mg/mL of phosphate buffer. Absorbance (OD_450_) was measured every minute for 30 min at 20 °C in a microplate reader (Infinity Pro200 spectrophotometer, Tecan, Spain). The lysozyme activity in the skin mucus samples was determined by comparing the reduction in absorbance to the prepared standard curve. The results were then expressed as IU/mg of protein.

TPA was measured following the procedure described by Moyano et al. (1996) and adapted for skin mucus by Sanahuja, et al. (2019a). Bovine trypsin (Sigma) was diluted in Milli-Q water to prepare a standard curve. Skin mucus samples and standard solutions were incubated for 30 min in 500 µL of 50 mM Tris–HCl pH 9.0 buffer containing 1% casein. Then, the reaction was immediately stopped by adding trichloroacetic acid (TCA, 12%). After being maintained at 4 °C for 1 h, the samples were centrifuged at 7500 × g for 5 min at 4 °C. Supernatants were recovered and absorbance was measured at 280 nm in a microplate reader (Infinity Pro200 spectrophotometer, Tecan, Spain). Each sample was analysed in triplicate. Controls were established by stopping reactions, adding TCA solution, before mixing with the casein buffer, and the absorbance was subtracted from those obtained after mixing with casein. Total alkaline protease activity was expressed as IU/mg of protein.

### Skin mucus and plasma antibacterial activity

Antibacterial activity of both skin mucus and plasma was tested against three main pathogens for rainbow trout, *Aeromonas hydrophila* (CECT 398), *Aeromonas salmonicida* (CECT 894), and *Yersinia ruckeri* (CECT 4319), and against *E. coli* (CECT 45), as non-pathogenic bacteria for fish, from the Spanish Type Culture Collection (CECT, University of Valencia, Valencia, Spain). Following the recommendations of the culture collection, *A. hydrophila, A. salmonicida*, and *E. coli* were routinely cultured in tryptic soy broth (TSB) or tryptic soy agar (TSA) (Laboratorios Conda, Spain), and nutrient broth (NB) or nutrient agar (NA) (Scharlau, Spain) were used for *Y. ruckeri.* Bacterial strains, stored at − 80 °C, were streaked on TSA or NA plates and incubated at 30 °C for 24 h. Several colonies of each strain were gently resuspended in 20 mL of TSB or NB and grown at 25 °C and 200 rpm for 16 h (ON). Each strain was transferred to 20 mL of fresh culture medium, at an initial cell density (OD) of 0.04, measured at 400 nm. The cultures were incubated under the same conditions as the initial growth (25 °C) until reaching the exponential growth phase (OD_400_ = 0.2).

To assess the antibacterial activity of mucus and plasma, three pools were created for each experimental condition (each pool consisted of samples from three different fish). Based on the results of an initial assay that demonstrated strong antibacterial activity of plasma against all bacterial strains tested, the plasma pools were diluted with Milli-Q water to a final concentration of 1.5 µg protein/mL. Adapting previous protocols from Sanahuja et al. (2019a) the antibacterial assay was performed as follows: aliquots of 50 µL of each bacterial suspension were incubated with 50 µL of fresh medium (TSB/NB 3X) and 100 µL of each sample pool (mucus or plasma). Triplicates of 100 µL of sample pools used were incubated with 100 µL of medium (TSB/NB 2X) as negative controls. In parallel, 50 μL of the same bacterial suspension was incubated with 150 μL of fresh medium (TSB/NB 1X), as positive controls (untreated control). As a contamination control, 200 µL of TSB or NB (1X) was incubated in triplicate, and its absorbance was subtracted from the positive bacterial growth control.

All incubations were carried out in a sterile flat-bottomed 96-well plate (Biolite Thermo Scientific), and bacterial growth was measured by absorbance at* λ* = 400 nm every 30 min for 14 h at 25 °C with a microplate reader (Infinity Pro200 spectrophotometer, Tecan, Spain).

Bacterial growth inhibition was also calculated at even hours of incubation as follows:

$$\%\;inhibition=\left(1-\frac{{OD}_{400}\;of\;(sample\;-\;negative\;control)}{{OD}_{400}\;of\;(untreated\;control\;-\;negative\;control)}\right)\times100$$

where “negative control” in the numerator refers to the control of mucus samples plus medium and in the denominator refers to medium contamination control.

### Statistical analysis

Results were expressed as mean ± standard error of mean (SEM). All data were checked for normality and homoscedasticity prior to their analysis. Statistical analysis was performed using Student’s *t*-test and differences were considered statistically significant at *p* < 0.05. A principal component analysis (PCA) was also carried out with both skin mucus and plasma biomarkers analysed in the different anaesthetic groups of fish. The two artificial variables accounting for the most variance were selected for plotting the results in two dimensions. All tests were conducted with SPSS software version 22.0 (IBM Corp, Armonk, NY, USA).

## Results

The time-lapse of the skin mucus sampling is recorded in Table 1. Whereas no differences were observed in the process of capture and transport of animals to the anaesthetic tanks, the study revealed significant differences in the time required for fish to reach full anaesthesia when comparing MS-222 and clove oil. MS-222 took approximately twice as long to induce full anaesthesia compared to clove oil (*p* < 0.05), at the specific concentrations of anaesthetics used in the study. Sampling times were extremely short for both treatments, and no significant differences were observed in the time required to collect mucus samples between the two anaesthetic treatments. However, the recovery time after anaesthesia was four times higher for clove oil than for MS-222. All procedures extended for 291 ± 21 s and 368 ± 36 s for MS-222 and clove oil (*p* < 0.05) respectively, indicating that for both treatments the time-lapse is fast and effective, even in farm conditions.

The analysis of mucus biomarkers and their ratios (Table 2) showed no significant differences between the anaesthetic treatments in terms of skin mucus composition, regarding soluble protein, glucose, lactate, and cortisol concentrations. Plasma biomarkers, classic markers of immediate stress response, were also measured (Table 3). The protein concentration was equivalent for both treatments whereas the clove oil group showed higher values of glucose (*p* > 0.05) and lactate (*p* < 0.05) resulting in a lower aerobic ratio (glucose/lactate) (Table 3). Accordingly, to the time of full anaesthesia, the MS-222 group showed increased plasma cortisol (almost twofold, *p* < 0.05) when compared to individuals immersed in a clove oil solution (Table 3).

**Table 2:** Skin mucus bioindicators after the anaesthetic treatment

	**MS-222**	**Clove Oil**
Protein (mg/mL)	4.02 ± 0.84	3.50 ± 0.75
Glucose (μg/mL)	15.68 ± 1.59	17.39 ± 1.46
Lactate (μg/mL)	3.88 ± 1.66	4.33 ± 1.12
Cortisol (ng/mL)	0.17 ± 0.004	0.16 ± 0.02
Ratio glucose/protein (μg/mg)	5.42 ± 0.97	5.99 ± 1.15
Ratio lactate/protein (μg/mg)	0.82 ± 0.22	1.07 ± 0.17
Ratio cortisol/protein (ng/mg)	0.05 ± 0.01	0.05 ± 0.01

Values are expressed as mean ± SEM (standard error of mean). Significant differences between treatments are indicated by * (Student’s *t*-test, *p* < 0.05)

**Table 3:** Response of plasma bioindicators to the anaesthetic treatment

	**MS-222**	**Clove oil**
Protein (mg/mL)	28.01 ± 1.11	29.37 ± 0.10
Glucose (mg/dL)	66.65 ± 2.29	78.80 ± 4.81
Lactate (mg/dL)	29.09 ± 3.51	36.16 ± 2.51*
Aerobic ratio (mg glucose/mg lactate)	2.51 ± 0.24	2.26 ± 0.19 *
Cortisol (ng/mL)	7.74 ± 1.98	4.45 ± 1.09 *

Values are expressed as mean ± SEM (standard error of mean). Significant differences between treatments are indicated by * (Student’s *t*-test, *p* < 0.05)

Regarding the defensive biomarkers in mucus, total antioxidant power (FRAPs), total protease (TPA) and lysozyme activity were measured and summarized in Table 4. Whereas total protease activity was not affected by the different anaesthetic treatments, clove oil led to higher antioxidant power and lysozyme activity in skin mucus when compared with MS-222 anaesthetized fish (*p* < 0.05).

**Table 4:** Response of skin mucus defensive biomarkers to the anaesthetic treatment

	MS-222	Clove oil
FRAPs (mmol/mg of protein)	0.34 ± 0.03	1.41 ± 0.27*
Total alkaline protease activity (IU/mg of protein)	49.98 ± 6.46	50.77 ± 10.08
Lysozyme activity (IU/mg of protein)	19.14 ± 1.17	29.24 ± 3.31*

Values are expressed as mean ± SEM (standard error of mean). Significant differences between treatments are indicated by * (Student’s *t*-test, *p* < 0.05)

**Fig. 1:**
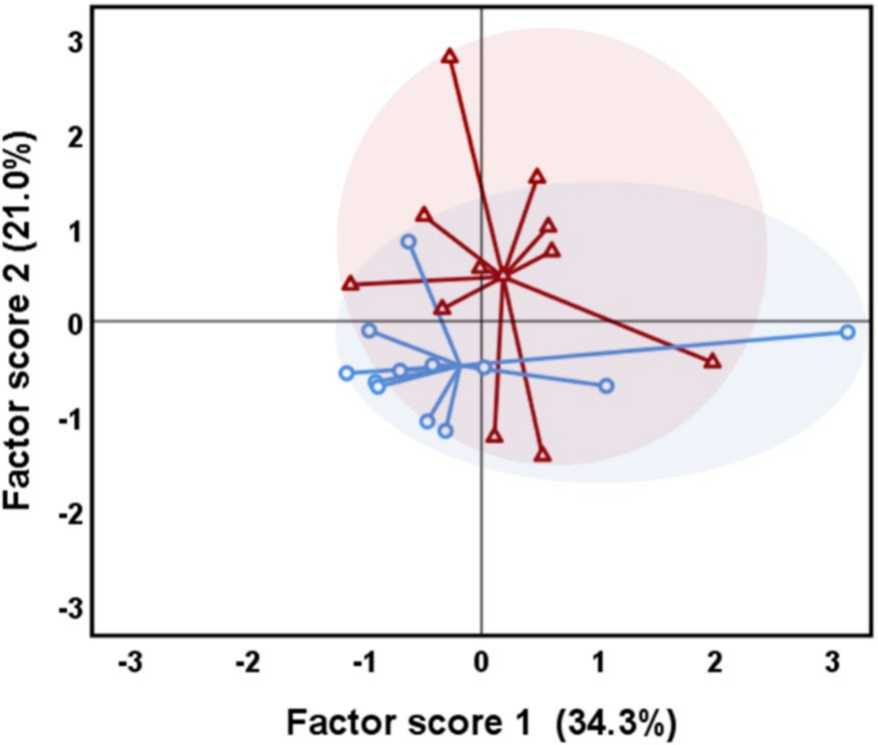
Plot of principal component analysis (PCA) representing the assembly of the stress and defensive biomarkers of the rainbow trout individuals anaesthetized with clove oil (red triangles) or MS222 (blue circles)

Figure 1 presents the dispersion and distribution of the two groups (MS222 and clove oil) in relation to the two principal components that account for the greatest variance in the PCA. These components explain 55.3% of the total variance and were derived based on both skin mucus and plasma biomarkers. The centroids of the two anaesthetics groups appear to be in proximity, located within the range of − 1 to 1 on both components, suggesting analogous effects in rainbow trout fish.

**Fig. 2:**
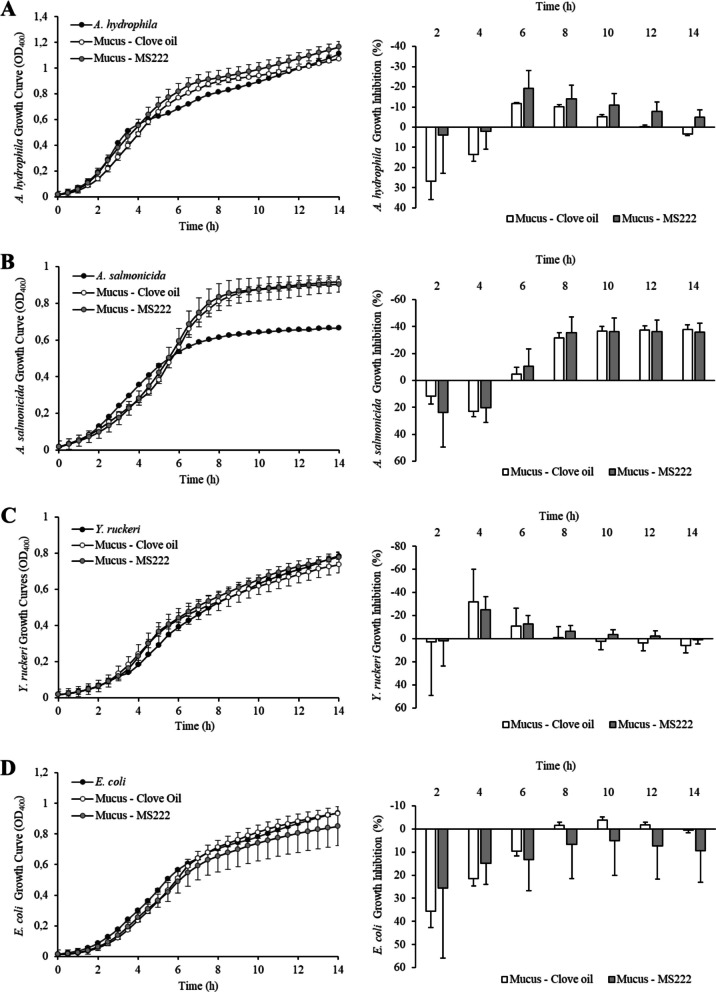
Bacterial growth inhibition by skin mucus of *O. mykiss* anaesthetized with clove oil (white) or MS-222 (grey). Values are expressed as mean ± SEM (standard error of mean). Significant differences between treatments are indicated by * (Student’s *t*-test, *p* < 0.05)

**Fig. 3:**
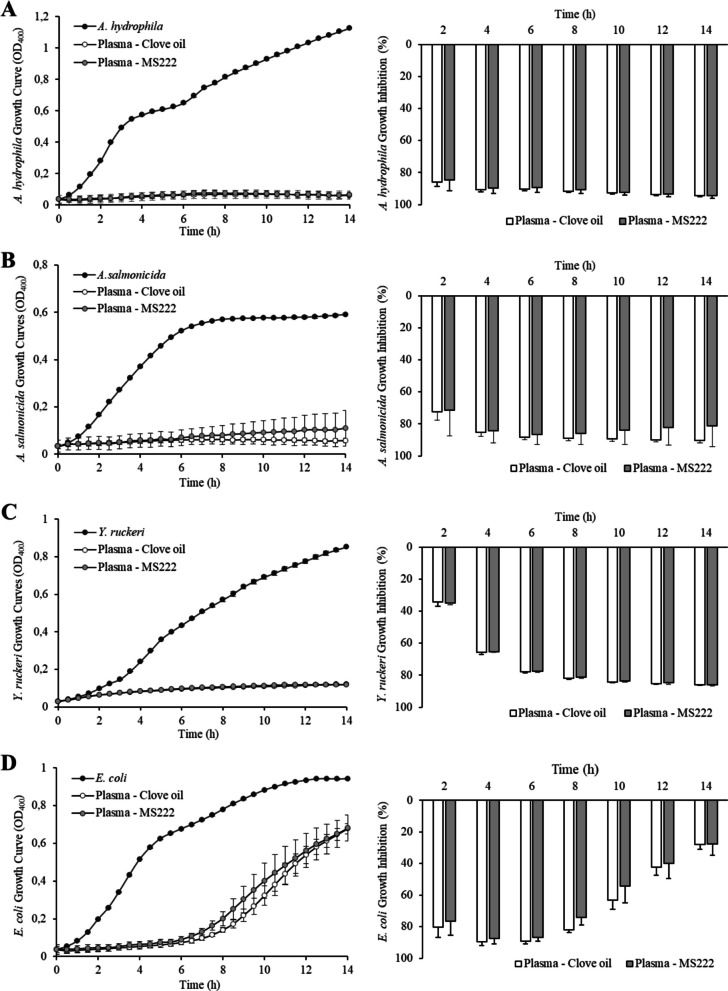
Bacterial growth inhibition by plasma of *O. mykiss* anaesthetized with clove oil (white) or MS-222 (grey). Values are expressed as mean ± SEM (standard error of mean). Significant differences between treatments are indicated by * (Student’s *t*-test, *p* < 0.05)

Antibacterial activity of skin mucus was determined against three pathogenic bacteria for rainbow trout: *A. hydrophila, A. salmonicida*,* and Y. ruckeri,* and a non-pathogenic strain of *E. coli*. Figure 2 shows the growth curve (left figures) of each bacterium without or with mucus from trout treated with either MS-222 or clove oil and the corresponding growth inhibition values (right figures). Rainbow trout skin mucus showed limited antibacterial activity against all the bacterial strains tested, being observable during the first hours of incubation (0–4 h). After prolonged incubation, the presence of mucus seemed to stimulate bacterial growth except for *E. coli* (Fig. 2D). The stimulatory effect was especially strong for *A. salmonicida* (Fig. 2B). Besides, no treatment effects were observed in the antibacterial activity of skin mucus for all the bacteria studied. Rainbow trout plasma strongly inhibited bacterial growth. Figure 3 shows both bacterial growth and plasma inhibition for the pathogenic strains used and for *E. coli*. Plasma inhibited almost completely the growth of the three rainbow trout pathogens, showing inhibition values over 80–90% (Fig. 3A, B, and C). In the case of *E. coli,* plasma antibacterial activity appeared to be reduced after prolonged incubation (Fig. 3D). Again, no significant differences existed in antibacterial activity between plasma samples obtained from fish subjected to the different anaesthetic treatments.

## Discussion

The present study assesses the feasibility of substituting MS-222 with clove oil as an anaesthetic for research applications in rainbow trout farming. It is important to highlight that the majority of comparative studies between MS-222 and clove oil and their effects on fish have been carried out under controlled laboratory settings, often using identical or similar anaesthetic concentrations. However, in fish farms, many uncontrollable variables could interact with anaesthesia efficacy or physiological effects. Considering the regulatory aspects, clove oil represents a better anaesthetic treatment option in European fish farms in terms of facilitating animal management. In this study, the clove oil dose was selected based on the concentrations routinely used in farm procedures for fish of similar size. Conversely, MS-222 concentration was decided according to published literature for rainbow trout of comparable size under laboratory conditions (Anderson et al. 1997; Holloway et al. 2004; Pounder et al. 2018; Topic Popovic et al. 2012). Furthermore, this study contributes to the development of a non-invasive tool to evaluate farm fish status or welfare, as it has been recently proposed for marine species (Fernández-Alacid et al. 2018; Sanahuja et al. 2019b).

Although clove oil anaesthesia in fish has been studied and proposed as a strong alternative to other chemical agents, in this work, we provide evidence supporting its widespread use on farms. Our results showed that MS-222 treatment required twice the time to induce full anaesthesia, while clove oil extended the recovery time by over 3 min in trout. These data are consistent with previously reported findings obtained under laboratory conditions (Anderson et al. 1997; Keene et al. 1998). Similar observations have been reported for other fish species, such as the red pacu (*Piaractus brachypomus*) (Sladky et al. 2001), zebra fish (*Danio rerio*) (Grush et al. 2004), cobia (*Rachycentron canadum*) (Gullian and Villanueva 2009), Senegalese sole (*Solea senegalensis*) (Weber et al. 2009), and pacific hagfish (*Eptatretus stoutii*) (McCord et al. 2020).

The difference in the recovery time may be attributed to the distinct effects these anaesthetics have on respiratory and heart rates. The increase in respiratory and heart rates described for MS-222 may facilitate a rapid elimination of the drug excess from the bloodstream, while the inhibitory effect of clove oil on the respiratory system results in a longer retention in the organism, resulting in a longer recovery period (Hisaka et al. 1986; Keene et al. 1998; Priborsky & Velisek 2018; Wagner et al. 2003). While this extended anaesthesia can be advantageous for certain procedures, it may also increase the risk of hypoxia and induce other physiological disturbances. Therefore, careful consideration of clove oil use is necessary, particularly in interventions requiring rapid recovery. Moreover, the lipophilic nature of clove oil facilitates rapid penetration through the gill epithelium and high absorption by body tissues, which, although beneficial for anaesthetic efficacy, could potentially result in prolonged presence and toxic effects, further delaying recovery. In contrast, the hydrophilic properties of MS-222 result in a rapid distribution throughout the body, followed by immediate elimination (Jia et al. 2022; Keene et al. 1998).

Although anaesthesia is commonly used to reduce stress in fish during routine procedures, it has been suggested that exposure to anaesthetic agents may itself act as a stressor. In this regard, anaesthesia with propofol and eugenol was shown to induce an increase in serum cortisol and glucose in *Nile tilapia* (*Oreochromis niloticus*) (Zahran et al. 2021). It has also been described that the use of MS-222 and clove oil causes an increase in blood glucose in red pacu (*P. brachypomus*), but no differences were found between the two anaesthetics (Sladky et al. 2001). In the present study, blood glucose and protein concentrations in plasma did not differ between the two anaesthetic treatments in rainbow trout, whereas MS-222 anaesthesia led to a higher cortisol concentration. The higher plasma cortisol found in the MS-222-anaesthetized group could be attributed to the longer time required to reach full anaesthesia, as fish may experience additional stress in the supplementary tanks. Other authors (Holloway et al. 2004; Velisek et al. 2011) have reported no differences in plasma glucose and cortisol when comparing anaesthesia with clove oil or MS-222 in trout. Notably, Holloway et al. (2004) also reported that anaesthetized fish exhibited lower values of both biomarkers compared to fish subjected to concussion, supporting the role of anaesthesia in mitigating stress.

Our data reveals higher values of lactate in plasma in trout individuals treated with clove oil. Since lactate is produced from the anaerobic glucose metabolism during hypoxic conditions, this increase could be attributed to the enhanced depressive effect of this anaesthetic on the respiratory system. Hence, Wagner et al. (2003) suggested that the longer recovery times associated with clove oil anaesthesia may contribute to higher plasma lactate levels observed during the recovery period in rainbow trout.

It is important to note that the current results were obtained using the anaesthetic doses assayed for MS-222 and clove oil, which correspond to the concentrations commonly used in laboratory settings and local trout farms, respectively. It is well known that different doses can influence fish lethargy, behavioural responses, and the associated physiological response (Anderson et al. 1997; Jia et al. 2022; Keene et al. 1998); therefore, extrapolation of these results to other conditions should be approached with caution.

Fish skin mucus is a complex fluid, continuously secreted to the external medium by metabolically active epidermic cells, which is composed of a wide range of molecules immersed in a matrix of mucins. Due to its strategic location, the non-invasive nature of sampling that does not imply invading the internal medium of fish, and the fact that its composition and properties undergo alterations in response to various fish conditions, the study of this biological component has gained significant attention in recent years. Numerous studies showed an increase in fish skin mucus of stress-associated biomarkers such as cortisol, glucose, and/or lactate, typically analysed in plasma, after stressing fish by netting (Fernández-Alacid et al. 2018), crowding (Guardiola et al. 2016), inducing hypoxia (De Mercado et al. 2018), or being exposed to water pollutants (Carbajal et al. 2019). Moreover, high correlations between plasma and mucus levels have been reported for some of these biomarkers (protein, glucose, and cortisol) in response to stressors (Fernández-Alacid et al. 2019b).

Limited research has been conducted on the effects of anaesthetic exposure on skin mucus. Our study revealed that none of the stress biomarkers analysed were affected during anaesthesia whether clove oil or MS-222 were used. The detected biomarker levels are similar to basal levels described in the literature for rainbow trout and far from those obtained when inducing stress (De Mercado et al. 2018; Franco-Martinez et al. 2022). These results are in accordance with other reports that found no changes in cortisol and glucose levels in discus (*Symphysodon aequifasciata*) skin mucus before and after a 10-min anaesthesia with MS-222 at different concentrations (Ouyang et al. 2020). The higher lactate concentration found in plasma for clove oil treatment, with respect to MS-222 treatment, was not observable in skin mucus. The presence of lactate in fish skin mucus can be explained by the anaerobic metabolism of epidermal cells and by the increased disposal of the molecule from the bloodstream when lactate levels in the blood rise significantly min. In normoxic fish, Ra lactate decreased from 18.2 to 13.1 molkg–1 min–1 and Rd lactate from 19.0 to 12.8. Ra and Rd were always matched, thereby maintaining a steady baseline blood lactate concentration of ~ 0.8mmoll–1. By contrast, the hypoxic fish increased blood lactate to 8.9mmoll–1 and Ra lactate from 18.4 to 36.5 molkg–1 min–1. This stimulation of anaerobic glycolysis was unexpectedly accompanied by a 52% increase in Rd lactate from 19.9 to 30.3 molkg–1 min–1. White muscle was the main producer of lactate, which accumulated to 19.2 mol g–1 in this tissue. This first study of non-steady-state lactate kinetics in fish shows that the increase in lactate disposal elicited by hypoxia plays a strategic role in reducing the lactate load on the circulation. Without this crucial response, blood lactate accumulation would double.10.1242/jeb.048512https://github.com/citation-style-language/schema/raw/master/csl-citation.json" (Omlin & Weber 2010). Correlation between plasma and skin mucus stress-associated biomarkers (cortisol, glucose, and lactate) has been described during stress (Fernández-Alacid et al. 2019b). Therefore, our results suggest that the two anaesthetics did not exert differential effects on epidermal anaerobic metabolism. Moreover, the increase in blood lactate observed in the clove oil group was not high enough to alter mucus composition, as typically occurs under acute stress conditions. Despite these considerations, recent studies on fish skin mucosa suggest the presence of a local production and regulation of stress-related hormones, such as cortisol, within the skin itself. This phenomenon, known as the cutaneous stress response system (CSRS), implies that mucus cortisol and other small molecules may provide unique insights into local skin stress (Gozdowska et al. 2022; Kulczykowska 2019; Pomianowski et al. 2023), rather than solely reflecting systemic stress. The absence of significant differences observed in the present study suggests that the anaesthetic agents used did not alter the CSRS response.

Although no significant differences in mucus stress biomarkers were observed between treatments, further studies analysing the temporal dynamics of these metabolites would be highly valuable for assessing the effects of anaesthetics at different stages. However, such detailed analyses are challenging to perform under field conditions.

Proteases contribute to fish skin protection by directly interfering with pathogen colonization and/or by activating other immune compounds (Guardiola et al. 2014; Soltanian et al. 2018; Subramanian et al. 2007). The results regarding immune skin mucus parameters showed that the skin mucus protease activity was not affected by the differential anaesthetic treatment. Other authors have described that clove oil produced higher protease activities in seabream mucus (Guardiola et al. 2016). Our results indicated that anaesthetizing rainbow trout individuals with clove oil instead of MS-222 does not compromise skin defensive status regarding protease-related functions.

In fish skin mucus, lysozyme responses may be induced rapidly and are not only related to bacterial recognition but also to other alarm or stress situations (Dash et al. 2018). In this work, we determined that the skin mucus from the clove oil anaesthetized group showed a higher lysozyme activity. The relevance of this enzyme activity through the anaesthetic process and recovery is not previously described and further assays relating to the rapid delivery of this enzyme under handling conditions are necessary.

MS-222 and clove oil anaesthesia have been described to alter the activity of various antioxidant enzymes (CAT, SOD, GST, GPx, and GR) in different tissues (Teles et al. 2019; Velisek et al. 2011). However, when using the FRAP method, the Fe^3+^-TPTZ reagent does not react, or reacts very slowly, with thiol groups of glutathione and proteins. As a result, the contribution of enzymatic antioxidant mechanisms to the FRAP value is negligible (Benzie & Devaki 2017). The elevated FRAP values observed in the clove oil-treated group (Table 4) likely reflect the inherent antioxidant properties of clove oil itself. Given that FRAP specifically quantifies non-enzymatic antioxidant capacity and considering that skin mucus exhibits delayed antioxidant responses to external stressors (De Mercado et al. 2018), the increased FRAP values in this study suggest a direct contribution of clove oil to the antioxidant potential of mucus during the experimental period. The antioxidant activity of clove oil has been extensively studied and reviewed (Ulanowska & Olas 2021) and is largely attributed to its main bioactive compound, eugenol. Eugenol is a phenolic compound composed of a single aromatic ring and a hydroxyl group, features that confer a strong capacity for free radical scavenging. The phenolic hydroxyl group can donate a hydrogen atom or an electron to stabilize free radicals, while the aromatic system facilitates the delocalization of unpaired electrons (Dai & Mumper 2010). Both clove oil and eugenol have been shown to effectively scavenge DPPH, ABTS, and superoxide anion radicals, neutralize hydrogen peroxide, inhibit lipid peroxidation, reduce Fe^3+^ (FRAP method), and chelate transition metals (Gülçin 2011; Gülçin et al. 2012). Therefore, the use of clove oil as an anaesthetic immersion drug may offer a less harmful or oxidative environment to fish compared to MS-222.

Testing bacterial growth inhibition in vitro using skin mucus and plasma, as standardized by Sanahuja et al. (2019a), provides a rapid, accessible method to evaluate fish innate immunity against bacterial pathogens. It is a realistic approximation of the defensive status, independently of the mechanisms or molecules involved (Guardiola et al. 2014). For this purpose, herein three main rainbow trout pathogens have been selected for their economic impact on farms: *Aeromonas hydrophila*, *Aeromonas salmonicida* and *Yersinia ruckeri*, and *Escherichia coli* as a non-pathogenic specie. Contrary to our previous studies on other marine fish species (Herrera et al. 2020; Sanahuja et al. 2019a), rainbow trout (*Oncorhynchus mykiss*) skin mucus demonstrated reduced antibacterial activity. This is a noteworthy finding due to the importance of skin mucus as a primary defence against pathogens. Although comparisons with previous studies are challenging due to differing methodologies, Hisar et al. (2014) also reported a lack of antibacterial activity in rainbow trout skin mucus against various human pathogens. Similarly, Abolfathi et al. (2022) found that rainbow trout mucus exhibited minimal or no activity against human pathogens such as *E. coli*, *B. subtilis*, and *S. aureus* but showed strong bactericidal activity against fish pathogens like *Vibrio harveyi*, *Y. ruckeri*, and *A. hydrophila*. Our current results demonstrated that the anaesthetic treatment did not alter the antibacterial properties of rainbow trout skin mucus for all the strains tested. Soltanian et al. (2018) obtained similar results when comparing the effects of different anaesthetic treatments on the antibacterial activity of rainbow trout skin mucus against *Y. ruckeri*. According to the literature available (Rainger & Rowley 1993; Zargari et al. 2018), rainbow trout plasma did show strong antibacterial activity. *E. coli* growth was the least affected by plasma when compared to the three rainbow trout pathogens, in accordance with Abolfathi et al. (2022) results for skin mucus.

The findings of this study indicate that clove oil is a viable agent for non-invasive monitoring of fish welfare. Principal component analysis (PCA) of skin mucus and plasma biomarkers revealed comparable physiological responses in rainbow trout to both anaesthetic agents evaluated. These results support the potential adoption of clove oil in aquaculture settings, particularly where regulatory frameworks permit the use of less toxic, naturally derived compounds. The integration of sustainable anaesthetics with minimally invasive sampling techniques demonstrates a promising approach to enhancing animal welfare. Notably, combining clove oil anaesthesia with skin mucus analysis represents a minimally invasive strategy that helps limit stress-induced physiological damage in fish. This approach may contribute to reducing mortality and minimizing economic losses associated with routine monitoring procedures.

## Summary

Although anaesthetic treatment in fish is often considered a stressor, it remains mandatory for any regulated procedure under European legislation. Our results indicate that clove oil is a strong alternative to MS-222 as an anaesthetic agent for use in rainbow trout farms and field studies. Clove oil anaesthesia resulted in a longer recovery time compared to MS-222, which should be considered when conducting in situ studies at fish farms. Most stress biomarkers in plasma and skin mucus were not significantly affected by the anaesthetic treatment, and clove oil even enhanced certain defensive biomarkers in skin mucus. As an authorized anaesthetic in Europe, clove oil emerges as a promising alternative for in situ aquaculture studies, offering a shorter withdrawal period and lower environmental impact. Therefore, when combined with minimally invasive mucus sampling, it allows for effective monitoring of fish health while ensuring recovery and return to the cage.

## Data Availability

Data are available from the authors upon reasonable request.
